# Zero Valent Iron Nanoparticle-Loaded Nanobentonite Intercalated Carboxymethyl Chitosan for Efficient Removal of Both Anionic and Cationic Dyes

**DOI:** 10.1021/acsomega.0c06251

**Published:** 2021-03-01

**Authors:** Abdelazeem S. Eltaweil, Ashraf M. El-Tawil, Eman M. Abd El-Monaem, Gehan M. El-Subruiti

**Affiliations:** Department of Chemistry, Faculty of Science, Chemistry, Alexandria University, P.O. Box 426, Alexandria 21321, Egypt

## Abstract

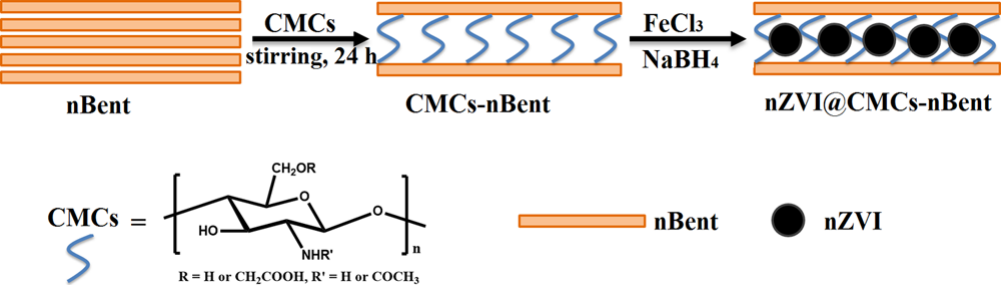

A zero valent iron-loaded nano-bentonite intercalated carboxymethyl chitosan (nZVI@nBent–CMC) composite was fabricated and characterized by FT-IR, TEM, TEM–EDX, XRD, BET surface area, and zeta potential measurements. The as-fabricated nZVI@nBent–CMC composite exhibited excellent removal efficiency for both anionic Congo red (CR) dye and cationic crystal violet (CV) dye. The maximum uptake capacities of CR and CV onto the nZVI@nBent–CMC composite were found to be 884.95 and 505.05 mg/g, respectively. The adsorption process of both dyes well fitted with the Langmuir isotherm model and pseudo-second order kinetic model. Thermodynamic data clarified that the adsorptions of both CR and CV onto the nZVI@nBent–CMC composite are spontaneous processes. Moreover, the adsorption of CR onto the nZVI@nBent–CMC composite was found to be an exothermic process while that of CV is an endothermic process. The nZVI@nBent–CMC composite also exhibited excellent reusability for both studied dyes without noticeable loss in the removal efficiency, suggesting its validity to remove both anionic and cationic dyes from wastewater.

## 1 Introduction

Although polymers are increasingly growing in many applications including electronics, packaging, food, and medicine, the majority of these polymers are nonbiodegradable.^1^ These nonbiodegradable polymers represent a great threat to the environment. Contrariwise, bio-based polymers such as cellulose, alginate, chitin, pectin, and gelatin are biodegradable and could be safely used in different applications.^2,3^ Among these biopolymers, chitosan is a natural polysaccharide polymer that is obtained by partial deacetylation of chitin and is considered as the second most abundant biopolymer after cellulose.^4,5^ Chitosan possesses special characteristics including hydrophilicity, biodegradability, biocompatibility, well adhesion properties, nontoxicity, and nonimmunogenicity, and it is a low-cost polymer.^6−8^ In addition, chitosan contains a variety of modifiable positions in its chain structure which facilitate its functionalization via N- and O-hydroxylation, carboxymethylation, polymer-grafting, and sulfonation to obtain chitosan derivatives with noteworthy features.^9^ Carboxymethyl chitosan (CMC), a chitosan derivative soluble in water which can be produced from the carboxylation of chitosan, having carboxymethyl substituents on some of the primary hydroxyl or/and amino sites of the glucosamine building units of the chitosan chain. CMC has excellent chelation and adsorption properties and can be utilized over a wide range of pH.^10^ CMC has garnered a great deal of interest owing to their amphoteric character, renewability, and their widespread applications in food preservation, drug delivery, cosmetics, biomedicine, and adsorption.^11,12^

Recently, many research studies highlight the fabrication of nanoclay-polymer composites because of their high thermal stability, good adsorptive properties, unique catalytic ability, high surface area, as well as low production cost.^13^ Among these nanoclays, nano-bentonite (nBent), which is an aluminum phyllosilicate constructed from two layers of tetrahedral silica sandwiching one layer of octahedral alumina.^14^ Till date, nanoclay-polymer composites have been applied in potential applications including catalysis, textiles, automotive, drug delivery, food packaging, and especially in wastewater treatment.^15^ Although, nanoclay-polymer composites are considered one of the most efficient adsorbent categories because of their high adsorption capacity, low cost, and high abundance in nature, they have a main drawback which is the difficulty to separate them after the adsorption process. Consequently, many research studies have reported the fabrication of the magnetic nanoclay-polymer composite.^16^ In general, centrifugation and filtration methods are used to separate the adsorbent material from aqueous solution.^17^ These applications are time-consuming and require extra cost.^18^ Compared with traditional centrifugation and filtration methods, the magnetic separation method is an efficient, fast, and economic method for the separation of magnetic adsorbents from the medium after the adsorption treatment of pollutants is completed.^19^ The separation of nonmagnetic adsorbents from the sample solution after the adsorption process is very difficult and also time-consuming. This problem can be solved by the incorporation of magnetic nanoparticles on the surfaces of nanocomposite adsorbents and then by using a magnet.^20^

Zero valent iron nanoparticles (nZVI) have garnered great interest for their environmental applications because of their excellent reactivity, low cost, biocompatibility, availability, easy of separation, and the existence of various reactive sites.^21^ Till now, there is great concern to fabricate air stable nZVI with low aggregation to retain their good adsorption and reduction properties. This can be achieved by using a stabilizer or by forming a composite that covers the nZVI surface, protecting them from exposure to air.^22,23^

One of the risks that poses a threat to humanity is dyes because of their toxic nature, nonbiodegradability, carcinogenicity, and high solubility in water.^24^ Despite these risks, there are many industries that strongly depend on dyes such as paper, textile, plastic, cosmetics, pharmaceuticals, and so forth.^25,26^ Therefore, various physical, chemical, and microbial techniques have been developed to removal dyes from wastewater.^27−29^ Among these techniques, the adsorption process is considered one of the most effective techniques to decontaminate various types of contaminants such as heavy metals, organic pollutants, and dyes.^30^ Therefore, seeking new bio-based and highly efficient adsorbents is the main goal at present.

In this scope, we aim to (i) develop a new magnetic biopolymer-based composite nZVI@nBent–CMC composite derived from the natural clay and biopolymer, (ii) characterize the developed magnetic nZVI@nBent–CMC composite by different tools, (iii) investigate the adsorptive efficiency and the adsorption phenomena of the developed magnetic nZVI@nBent–CMC composite in the removal of both Congo red and crystal violet as a models for anionic and cationic dyes, and, (iv) finally, study the reusability of the developed nZVI@nBent–CMC composite utilizing its magnetic properties for easy regeneration.

## 2 Results and Discussion

### 2.1 Characterization of the nZVI@nBent–CMC Composite

#### 2.1.1 FTIR Analysis

**Figure 1:**
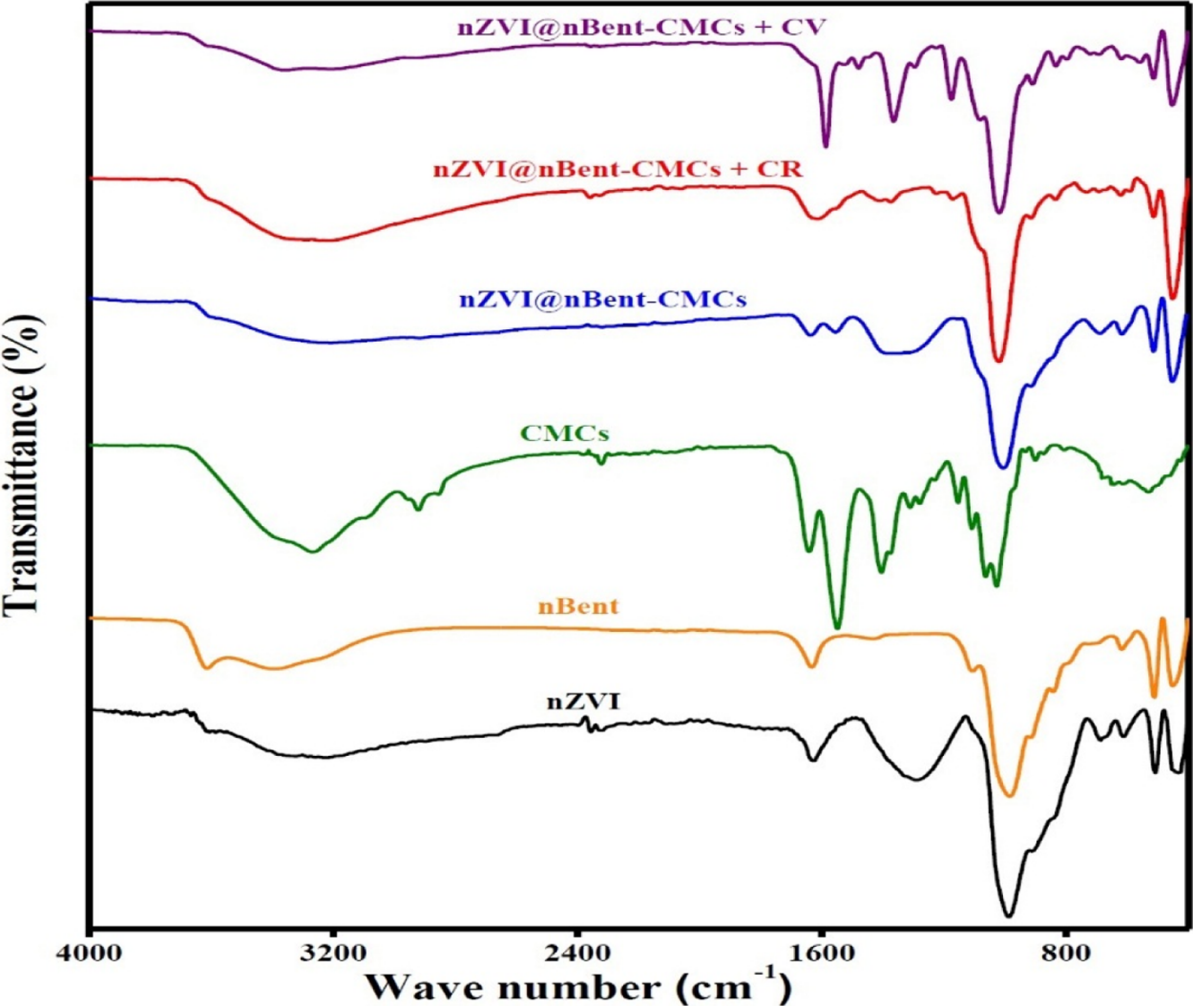
FTIR spectra of nZVI, nBent, CMC, and nZVI@nBent–CMC composite before and after adsorption of CR and CV dyes.

FTIR spectra of nZVI, nBent, CMC, and nZVI@nBent–CMC composite before and after adsorption of both CR and CV dye are depicted, as shown in Figure 1. For nZVI, the two peaks at 453 and 634 cm^–1^ are ascribed to Fe–O stretching vibration as well as the appearance of the discriminative peak of nZVI at 692 cm^–1^.^22^ The two peaks at 995 and 1320 cm^–1^ are assigned to the formation of FeOOH on Fe^0^. Furthermore, the broad peak around 3100 cm^–1^ is corresponded to −OH vibration stretching, and the peak at 1600 cm^–1^ is assigned to the −OH bending mode.^31^ For nBent, the peaks at 450 and 511 cm^–1^ are assigned to Si–O–Si and Si–O–Al bending vibration, respectively.^32^ Besides, the peak at 841 cm^–1^ is ascribed to the Al–Ca–OH bending vibration, and the strong peak at 990 cm^–1^ is attributed to the Si–O stretching vibration.^33^ Furthermore, the two peaks at 1633 and 3394 cm^–1^ are corresponding to H_2_O molecules on the surface of nBent, and the peak at 3614 cm^–1^ is attributed to the structural OH vibration.^34^ The FTIR spectrum of CMC shows two peaks at 918 and 1072 cm^–1^ corresponding to C–O stretching and C–O–C vibration, respectively.^11^ Additionally, the peak at 1329 cm^–1^ is ascribed to C–OH stretching vibration.^35^ The two peaks at 1420 and 1602 cm^–1^ are assigned to symmetric and asymmetric stretching vibrations of −COOH, respectively.^36^ Moreover, the peak at 2932 cm^–1^ is attributed to C–H stretching and the peak at 3447 cm^–1^ is assigned to the overlapping of N–H and O–H stretching vibrations. The FTIR spectrum of the nZVI@nBent–CMC composite obviously demonstrates the main peaks of the composite components. In addition, there is a strong peak at 1005 cm^–1^ which results from the overlap of CMC, nBent, and nZVI.^37^ Hence, FTIR spectra confirm that the nZVI@nBent–CMC composite possesses plenty of function groups that make it an efficient adsorbent for the removal of anionic as well as cationic dyes from water. The FTIR spectrum of the nZVI@nBent–CMC composite with CR dye shows a peak at 1175 cm^–1^, which is assigned to SO_3_ stretching of CR dye. Moreover, the FTIR spectrum of the nZVI@nBent–CMC composite with CV dye clearly shows new two peaks compared with the virgin nZVI@nBent–CMC composite spectrum; the first peak at 1175 cm^–1^ is corresponding to C–N stretching vibration of CV dye, while the other peak at 1502 cm^–1^ is assigned to the tri-phenylmethane dyes. Furthermore, the existence of peaks between 1500 and 500 cm^–1^ is ascribed to the mono and para-di substituted benzene rings of CV dye.^38^

#### 2.1.2 TEM and TEM–EDX Analysis

**Figure 2:**
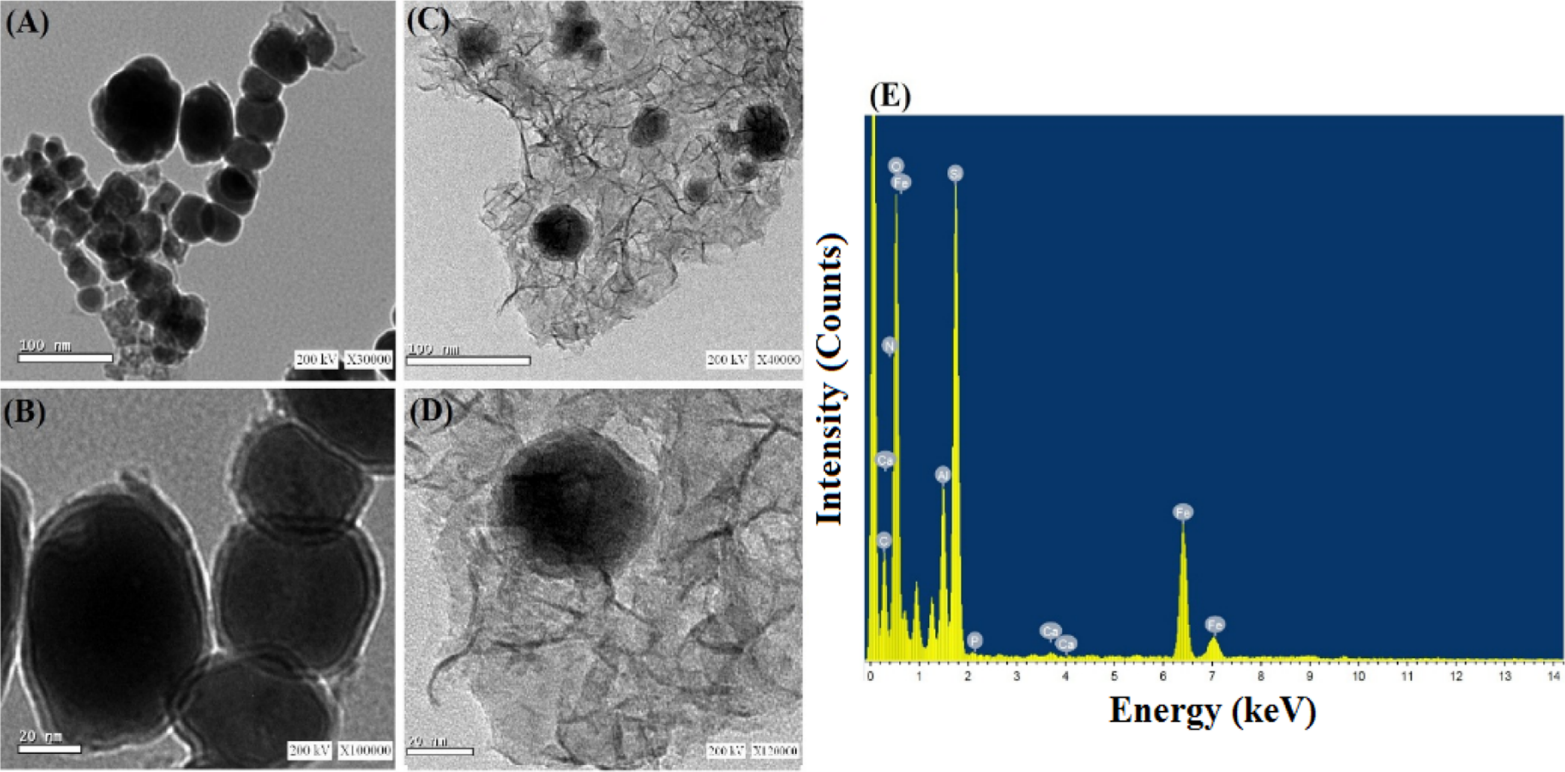
TEM images of nZVI (A,B), nZVI@nBent–CMC composite (C,D), and EDX spectrum of the nZVI@nBent–CMC composite (E).

Figure 2 shows the TEM images of the nZVI@nBent–CMC composite and nZVI. For nZVI, the TEM image (Figure 2A) clarifies the usual chain-like structure of nZVI particles.^39^ Furthermore, HR-TEM (Figure 2B) reveals the core–shell structure of nZVI that was confirmed previously in literature^40^ and suggested by FTIR results, as the nZVI particles consist of a core from Fe^0^ encompassed by a shell from FeOOH. On the other hand, TEM images of the nZVI@nBent–CMC composite (Figure 2C,D) demonstrate an intercalated structure of nZVI@nBent–CMC with almost spherical nZVI particles with no aggregation, revealing the well dispersion of nZVI in the intercalated structure.^41^ Besides, TEM–EDX was used to confirm the elemental composition of the fabricated nZVI@nBent–CMC composite. TEM–EDX analysis demonstrates that the main elements in the nZVI@nBent–CMC composite are carbon, oxygen, silicon, aluminum, iron, calcium, and a small trace of phosphorous (Figure 2E).

### 2.1.3 X-Ray Diffraction

**Figure 3:**
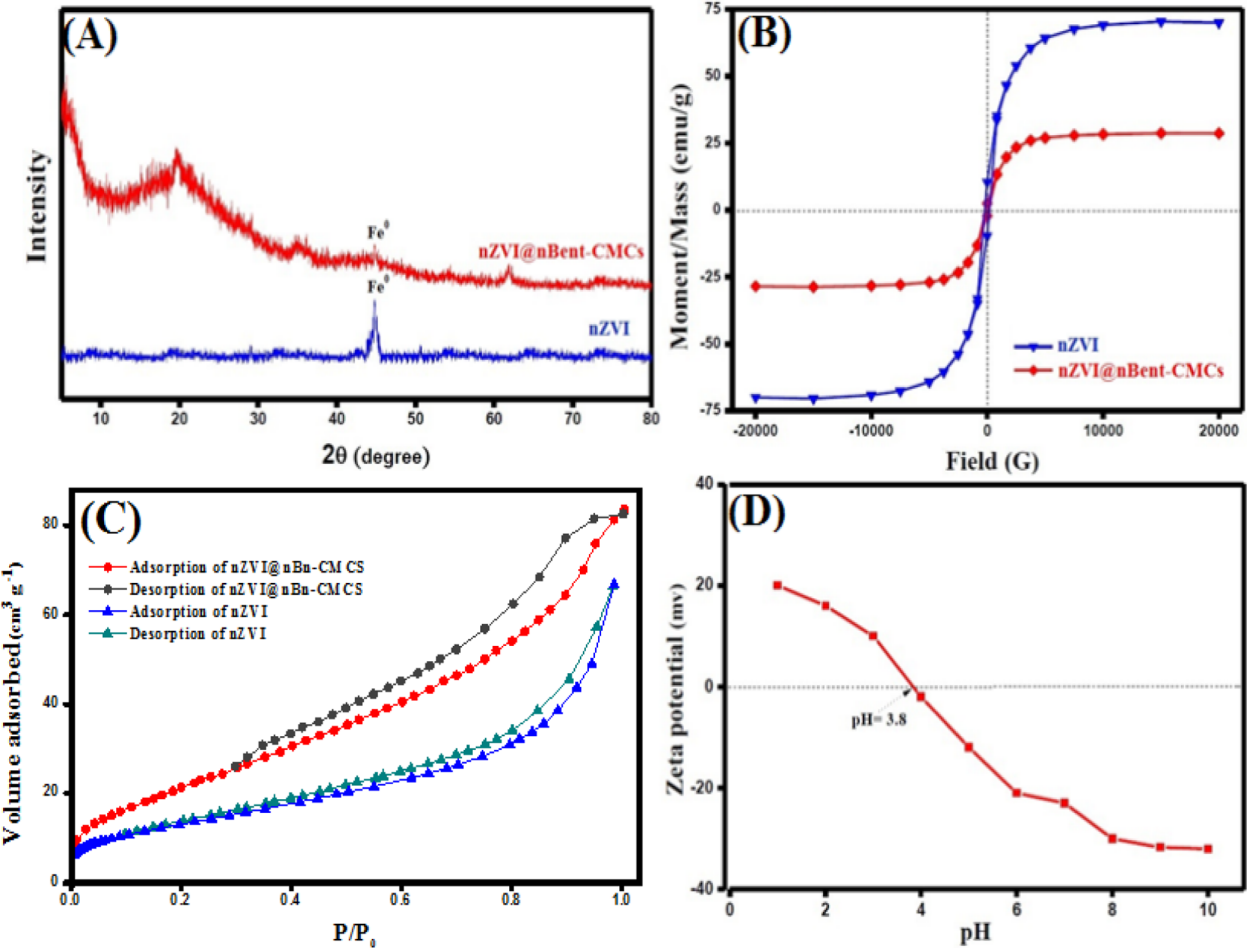
XRD patterns (A), hysteresis loop at room temperature (B), N_2_ adsorption–desorption isotherms for nZVI and nZVI@nBent–CMC composites (C), and zeta potential vs pH for the nZVI@nBent–CMC composite (D).

Figure 3A represents the X-ray diffraction (XRD) patterns of the nZVI and nZVI@nBent–CMC composite. The XRD pattern of nZVI reveals the discriminative peak of body-centered cubic nZVI at 2θ = 44.8°.^39^ Also, the nZVI@nBent–CMC composite pattern shows this distinctive peak of nZVI without the appearance any peaks for other iron oxides, which could be attributed to the complete coverage of nZVI with nBent–CMC that protects its surface from oxidation.^42^ Furthermore, nBent was recognized by the appearance of the distinguishing peaks at 2θ = 35 and 61.8°,^33^ whereas CMC was identified by the occurrence of a characteristic peak at 2θ = 21.1°.^36^ Thus, XRD patterns show the main components quite evidently, which confirms that the nZVI@nBent–CMC composite has been successfully synthesized.

#### 2.1.4 Magnetic Properties

The magnetic property of the nZVI@nBent–CMC composite was studied and compared to that of nZVI by utilizing VSM. The soft ferromagnetic behavior of both the nZVI@nBent–CMC composite and nZVI can be obviously seen from their hysteresis loops (Figure 3B), as their coercivity (*H*_c_) are 50.26 and 25.39 *G*, respectively. Additionally, there is a sharp decrease in the saturation magnetization (*M*_s_) of the nZVI@nBent–CMC composite (28.78 emu/g) compared to nZVI (72.5 emu/g), which is attributed to the presence of nonmagnetic nBent-intercalated CMC.

#### 2.1.5 BET Analysis

Figure 3C depicts the N_2_ adsorption–desorption isotherm curves for the nZVI@nBent–CMC composite and nZVI. Isotherms showed that nZVI exhibits a type II isotherm with *S*_BET_, total pore volume, and pore diameter of 35.62 m^2^/g, 0.149 cm^3^/g, and 1.49 nm, respectively. However, the nZVI@nBent–CMC composite exhibits a type IV isotherm with *S*_BET_, total pore volume, and pore diameter of 83.26 m^2^/g, 0.22 cm^3^/g, and 6.04 nm, respectively. The noticeable increase in *S*_BET_ of the nZVI@nBent–CMC composite compared with nZVI may be because of the high surface area of nBent and the good dispersion of nZVI in the nBent-intercalated CMC framework.^43^

#### 2.1.6 Zeta Potential

Figure 3D reveals the effect of pH on the zeta potential of the nZVI@nBent–CMC composite. It was found that the pH_PZC_ of the nZVI@nBent–CMC composite is 3.8, so the nZVI@nBent–CMC composite possesses a positive surface charge at pH < 3.8, which is most likely due to the protonation of amino groups in the CMC structure, while at pH > 3.8, the nZVI@nBent–CMC composite has a negative charge, which is because of the deprotonation of carboxyl groups. In light of this result, a strong Columbic interaction is expected between the nZVI@nBent–CMC composite and both anionic CR and cationic CV dye depending on the operating pH value which makes the nZVI@nBent–CMC composite an excellent candidate for the removal of both cationic and anionic contaminants.

### 2.2 Investigation of the Optimum Adsorption Conditions

#### 2.2.1 Effect of Contact Time

**Figure 4:**
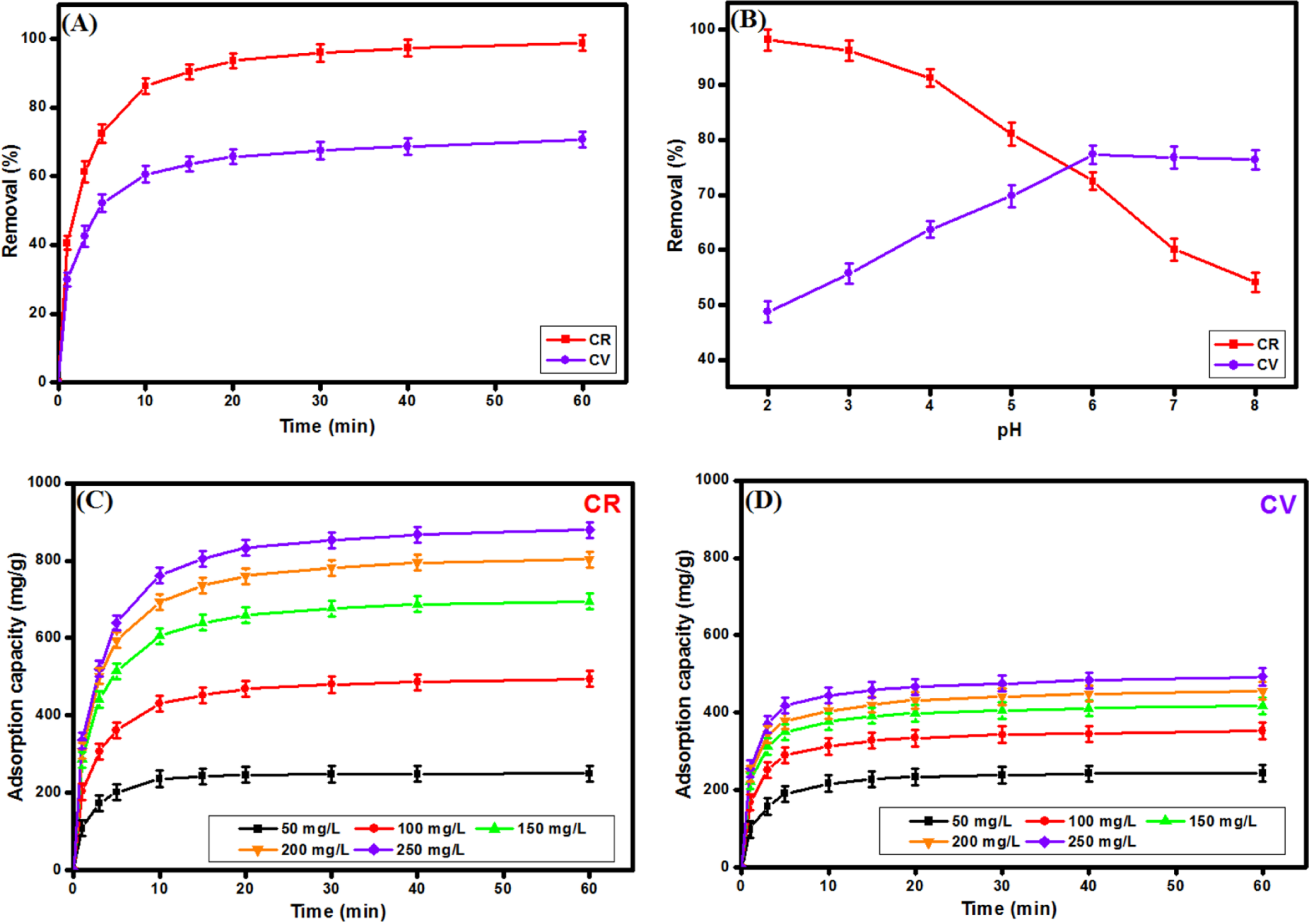
Effect of contact time (A), pH (B) on the removal efficiency of CR and CV onto the nZVI@nBent–CMC adsorbent [100 mL dye, 100 mg/L, 20 mg nZVI@nBent–CMC and 25 °C], uptake capacity of CR (C) and CV (D) onto the nZVI@nBent–CMC adsorbent [100 mL dye, 20 mg nZVI@nBent–CMC and 25 °C].

The effect of contact time on the adsorption of CR and CV was performed by soaking 20 mg of the nZVI@nBent–CMC composite in 100 mL of each dye solution (initial concentration = 100 mg/L) at 25 °C with continuous stirring (250 rpm) for 60 min. Figure 4A shows rapid adsorption at the beginning for both CR and CV onto the nZVI@nBent–CMC composite followed by a slow rate of adsorption till equilibrium. The removal efficiency of CR and CV onto the nZVI@nBent–CMC composite at equilibrium reached 98.8 and 70.7%, respectively. The fact that the removal efficiency of CR onto the nZVI@nBent–CMC composite is higher than that of CV could be because of the stronger electrostatic interactions between the CR and nZVI@nBent–CMC composite surface as well as the hydrogen bond possibility in the case of CR rather than CV.

#### 2.2.2 Effect of pH

It is well known that the removal of the dye is greatly affected by the pH of solution, because pH controls the charges on the surface of the adsorbent as well as the degree of dye ionization. In our study, the effect of pH on CR and CV uptakes has been studied at a pH ranging from 2 to 8 (Figure 4B). The obtained results reflected that there is a decrease in the CR removal efficiency with increasing pH. However, there is an increase in CV removal efficiency with increasing pH. This finding is in a good agreement with the obtained from zeta potential results for the nZVI@nBent–CMC composite. At low pH values (pH < 3.8) the nZVI@nBent–CMC composite as a positive surface charge enables stronger interactions with anionic contaminants (CR dye). Contrariwise, at high pH values (pH > 3.8), the nZVI@nBent–CMC composite as a negative surface charge shows a high affinity to adsorb cationic contaminants (CV dye).^44^ The optimum pH value for CR was found to be 2, while for CV the optimum pH value was found to be 6.

#### 2.2.3 Effect of Initial Concentration of Dye

It can be noticed from Figure 4C,D that the increase in the initial dye concentration from 50 to 250 mg/L causes an increase in the uptake capacity of CR and CV onto the nZVI@nBent–CMC composite from 249.35 and 243.63 to 879.58 and 492.80 mg/g, respectively. This behavior can be explained by the increase in the initial concentration which increases the driving force of dye from the bulk solution to the adsorbent surface, accordingly the adsorption capacity of both dyes onto the nZVI@nBent–CMC composite increases.^45^

### 2.3 Adsorption Isotherms

The adsorption isotherms show how the adsorbate molecules distribute between the bulk solution and the adsorbent surface at equilibrium. Therefore, the adsorption isotherm study is an important part for designing the adsorption system. In this study, the equilibrium data for adsorption of CR and CV onto the nZVI@nBent–CMC composite were analyzed by Langmuir and Freundlich models. The linear forms of Langmuir and Freundlich models are represented by the following equations^46^
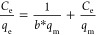
1
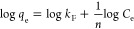
2herein, *C*_o_ and *C*_e_ symbolize the concentration of dye at zero time and at equilibrium, respectively. *q*_e_ symbolizes the uptake capacity on the nZVI@nBent–CMC composite surface at equilibrium and *q*_m_ represents the theoretical maximum uptake capacity of dye, while *b* (L/mg) represents the Langmuir constant, *n* and *k*_F_ [(mg/g) (L/mg)^1/*n*^] represent Freundlich constants.

**Figure 5:**
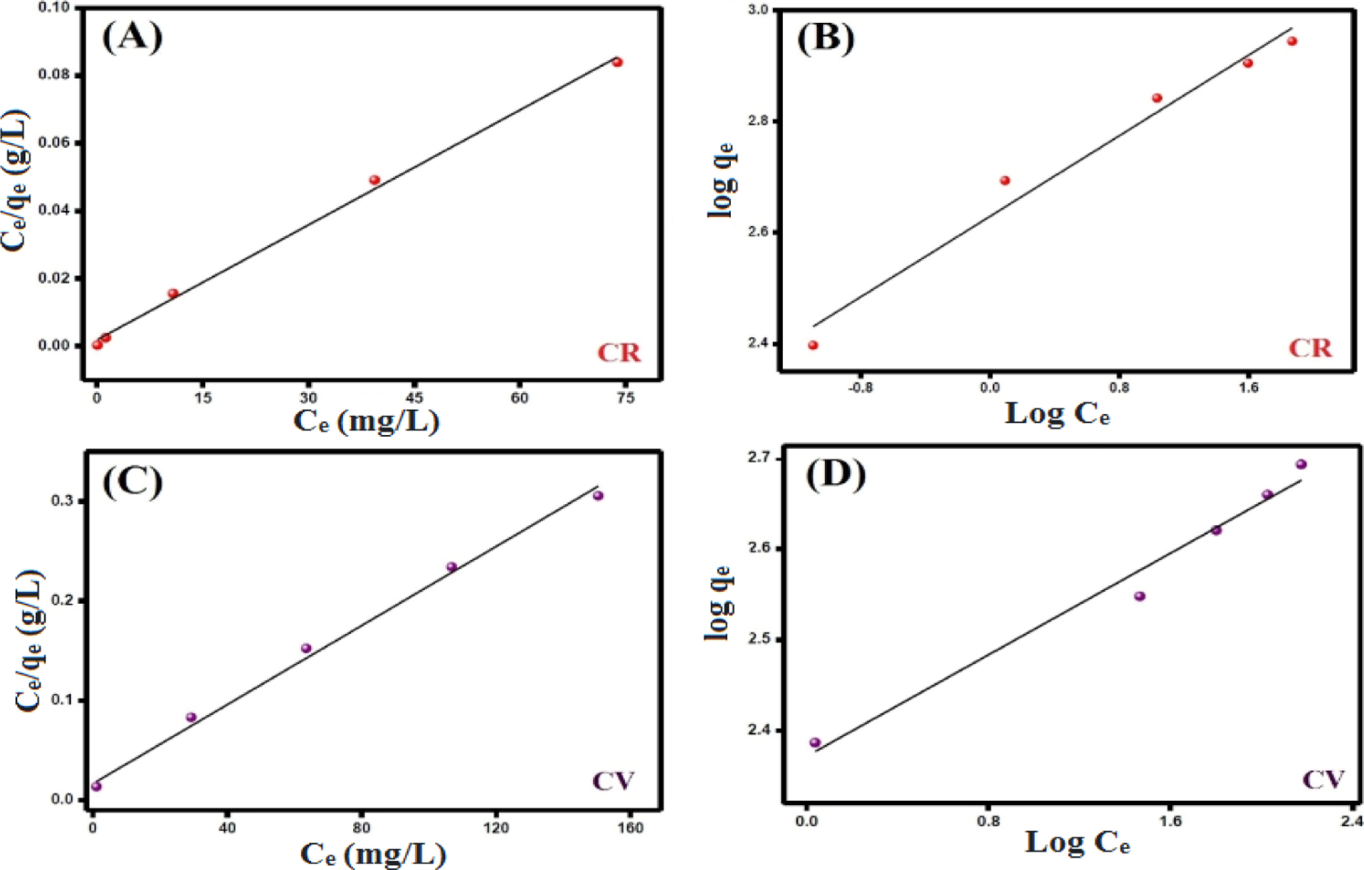
Langmuir and Freundlich isotherms of CR (A,B) and CV (C,D) onto the nZVI@nBent–CMC composite [*V* = 100 mL; *C*_o_ = 50–250 mg/L; *m* = 20 mg; *T* = 25 °C; pH_CR_ = 2; pH_CV_ = 6].

Figure 5 represents the linear plots of the Langmuir and Freundlich isotherms for both CR and CV. It was found from the determination coefficient values (*R*^2^) listed in Table 1 that the adsorption of both CR and CV onto the nZVI@nBent–CMC composite is more consistent with Langmuir’s model. Also, the Freundlich constant for both adsorption processes confirms the favorability of adsorption of these dyes onto the nZVI@nBent–CMC composite. In the literature, a similar isotherm model fitting has been obtained for the adsorption isotherms of various pollutants onto different adsorbents.^47,48^

**Table 1:** Adsorption Isotherm Parameters for the Adsorption of CR and CV Onto the nZVI@nBent–CMC Composite

	Langmuir			Freundlich		
model parameters	*q*_m_ (mg/g)	*b* (L/mg)	*R*^2^	*k*_F_ [(mg/g) (L/mg)^1/*n*^]	*n*	*R*^2^
**CR**	884.95 ± 7.89	1.156 ± 0.005	0.999	1.003 ± 0.051	6.121 ± 0.093	0.926
**CV**	505.05 ± 5.08	0.636 ± 0.002	0.993	0.984 ± 0.009	3.355 ± 0.022	0.748

### 2.4 Adsorption Kinetics

For predicting the CR and CV uptake rate onto the nZVI@nBent–CMC composite with respect to different initial concentrations of both dyes, kinetics study was carried out by applying a pseudo first order, pseudo second order, and intraparticle diffusion models. These kinetic models can be defined by the following equations^49^
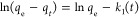
3
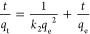
4
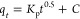
5where, *q*_*t*_ and *q*_e_ are the dye uptake capacities at time *t* and equilibrium, respectively. *k*_1_ (min^–1^) and *k*_2_ (g mg^–1^ min^–1^) are the rate constants of pseudo-first order and pseudo-second models, respectively. *K*_p_ (mg g^–1^ min^–0.5^) is the intraparticle diffusion constant and *C* provides an idea about the thickness of the boundary layer.

**Figure 6:**
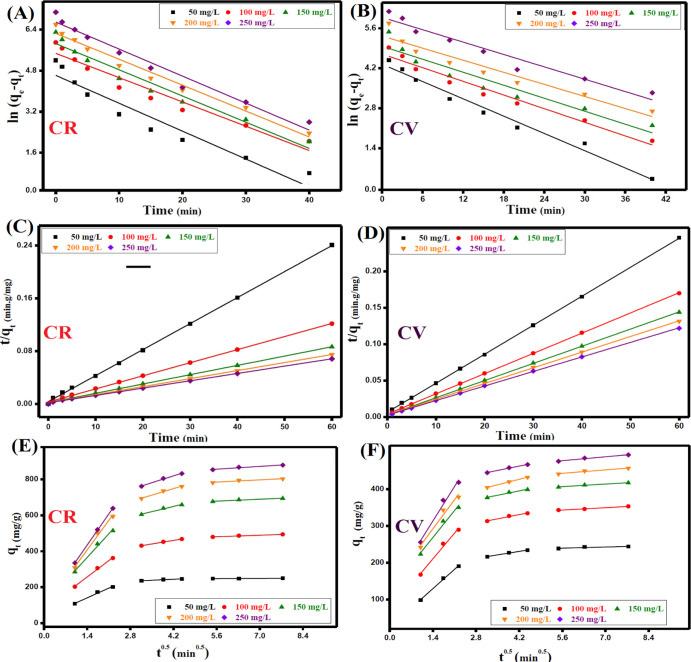
Kinetic plots for the adsorption of CR and CV onto the nZVI@nBent–CMC composite: (A,B) pseudo-first order, (C,D) pseudo-second order, and (E,F) intraparticle diffusion.

Figure 6A–D demonstrates the linear plots of pseudo-first order and pseudo-second models for the adsorption of CR and CV onto the nZVI@nBent–CMC composite. The obtained results (Table 2) indicate that the adsorption of both CR and CV onto the nZVI@nBent–CMC composite obeys pseudo-second order, where *R*^2^ values are closer to unity. In addition, there is a good convergence between the theoretical *q*_e,cal_ values obtained from the pseudo-second order model and those experimentally determined *q*_e,exp_. This result is similar to the previous kinetic results obtained for various adsorbent–adsorbate systems.^50,51^

**Table 2:** Outcome Kinetic Parameters from Pseudo-first Order and Pseudo-second Order Model for the Adsorption of CR and CV Onto the nZVI@nBent–CMC Composite

			pseudo first order			pseudo second order		
dye	*C*_o_ mg/L	*q*_e,exp_	*q*_e,cal_, mg/g	*k*_1_, min^–1^	*R*^2^	*q*_e,cal_, mg/g	*k*_2_, g mg^–1^ min^–1^	*R*^2^
**CR**	50	249.35	101.09 ± 1.42	0.109 ± 0.019	0.909	253.81 ± 2.32	0.00454 ± 0.017	0.999
	100	493.80	239.85 ± 2.02	0.095 ± 0.011	0.941	497.34 ± 2.98	0.00141 ± 0.008	0.998
	150	694.48	350.72 ± 2.79	0.102 ± 0.008	0.957	692.71 ± 3.09	0.00152 ± 0.007	0.997
	200	802.61	523.21 ± 3.59	0.101 ± 0.005	0.977	805.64 ± 3.91	0.00085 ± 0.005	0.998
	250	879.58	812.40 ± 5.01	0.105 ± 0.006	0.967	887.72 ± 4.11	0.00073 ± 0.002	0.999
**CV**	50	243.63	76.71 ± 1.01	0.101 ± 0.006	0.978	247.84 ± 2.15	0.00257 ± 0.011	0.997
	100	352.93	109.95 ± 1.51	0.079 ± 0.009	0.963	353.23 ± 2.58	0.00211 ± 0.008	0.999
	150	417.03	144.03 ± 1.85	0.075 ± 0.008	0.902	417.91 ± 2.75	0.00214 ± 0.007	0.999
	200	456.40	206.44 ± 2.29	0.070 ± 0.006	0.919	461.33 ± 2.81	0.00171 ± 0.005	0.999
	250	492.80	399.41 ± 2.74	0.072 ± 0.007	0.942	495.73 ± 3.08	0.00156 ± 0.004	0.999

Figure 6E,F clearly indicates that the adsorption of both CR and CV onto the nZVI@nBent–CMC composite occurs in three steps; (1) diffusion of dye molecules from the bulk solution to the external surface of the nZVI@nBent–CMC composite (*K*_p1_); (2) diffusion of the dye molecules to the pores of the nZVI@nBent–CMC composite (*K*_p2_); (3) diffusion of dye molecules into the interior pores of the nZVI@nBent–CMC composite until it reaches equilibrium (*K*_p3_).

It is clear from the *K*_p_ values for both CR and CV (Table 3) that *K*_p1_ > *K*_p2_ > *K*_p3_, which is most likely because of the change in the rate of diffusion of dye molecules during the three steps. Further, the noticeable increase in *K*_p_ values with the increase in the initial concentration for both dyes could be attributed to the increase in the driving force that results from the increasing of dye concentrations. Moreover, the *C* values and the short adsorption equilibrium time (about 20 min) suggest that the intraparticle diffusion step is not the rate-controlling step.^52^

**Table 3:** Outcome Kinetic Parameters from the Intraparticle Diffusion Model for CR and CV Adsorption of Onto the nZVI@nBent–CMC Composite

		first step			second step			third step		
dye	*C*_o_ mg/L	*K*_p,1_ mg g^–1^ min^–1/2^	*C*_1_	*R*^2^	*K*_p,2_ mg g^–1^ min^–1/2^	*C*_2_	*R*^2^	K_p,3_ mg g^–1^ min^–1/2^	*C*_3_	*R*^2^
**CR**	50	77.11 ± 0.35	33.12 ± 0.12	0.973	0.909 ± 0.10	8.01 ± 0.05	0.934	0.69 ± 0.02	243.99 ± 0.92	0.997
	100	129.55 ± 0.42	75.95 ± 0.28	0.992	0.941 ± 0.08	28.21 ± 0.10	0.997	6.13 ± 0.01	446.59 ± 2.11	0.976
	150	187.37 ± 0.63	103.69 ± 0.41	0.981	0.957 ± 0.08	41.48 ± 0.22	0.973	7.56 ± 0.04	636.85 ± 3.21	0.886
	200	233.88 ± 0.89	80.72 ± 0.30	0.982	0.977 ± 0.09	51.73 ± 0.32	0.978	8.83 ± 0.05	735.57 ± 4.74	0.815
	250	246.56 ± 0.97	89.89 ± 0.33	0.999	0.967 ± 0.07	54.39 ± 0.33	0.987	11.08 ± 0.08	794.85 ± 4.93	0.920
CV	50	74.70 ± 0.31	24.87 ± 0.09	0.993	13.38 ± 0.09	174.28 ± 0.53	0.991	2.25 ± 0.11	226.61 ± 0.83	0.642
	100	99.71 ± 0.38	70.83 ± 0.18	0.975	16.38 ± 0.12	261.72 ± 0.66	0.964	4.53 ± 0.13	317.55 ± 1.96	0.981
	150	104.28 ± 0.40	122.57 ± 0.39	0.968	16.95 ± 0.11	323.82 ± 0.82	0.961	5.07 ± 0.15	378.00 ± 2.27	0.958
	200	112.46 ± 0.41	134.59 ± 0.75	0.944	21.14 ± 0.13	337.62 ± 0.91	0.998	6.62 ± 0.17	405.67 ± 2.51	0.942
	250	133.48 ± 0.43	126.55 ± 0.43	0.969	16.86 ± 0.15	391.57 ± 0.99	0.983	7.59 ± 0.19	434.50 ± 2.73	0.958

### 2.5 Effect of Temperature and Thermodynamic Studies

**Figure 7:**
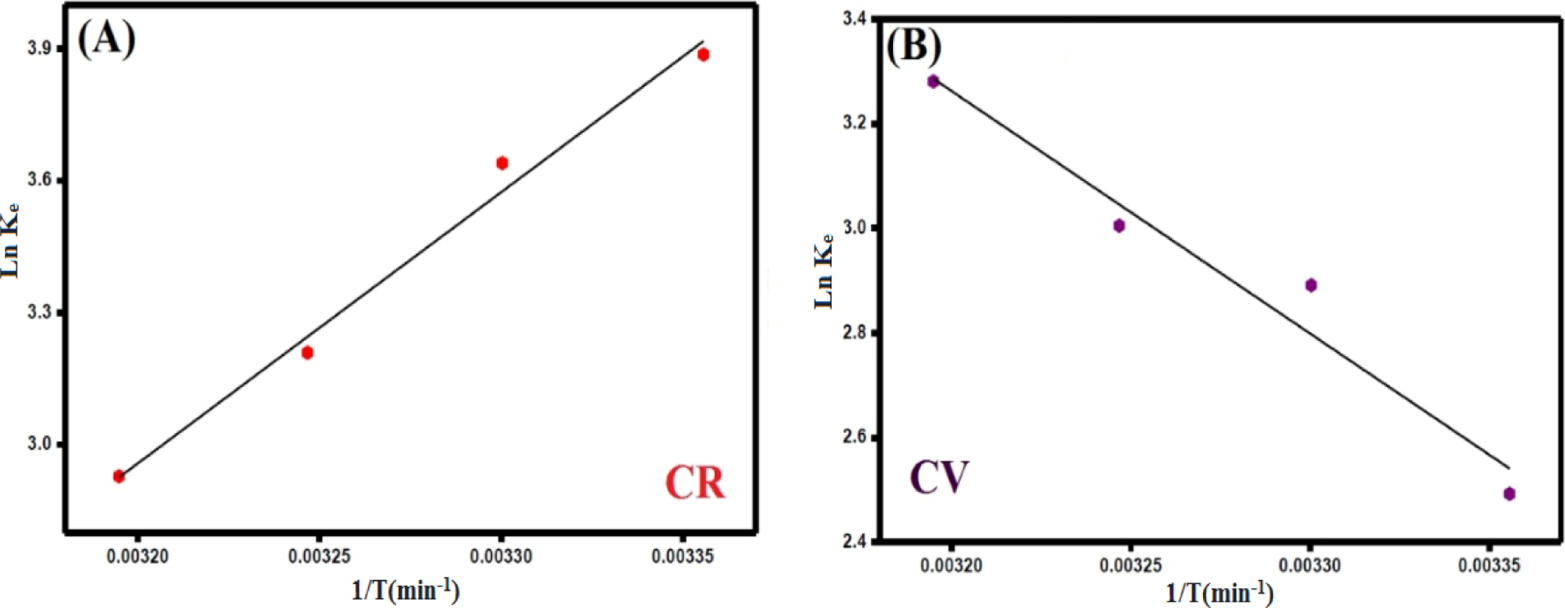
Van’t Hoff plot for the adsorption of CR (A) and CV (B) onto the nZVI@nBent–CMC composite.

The effect of temperature on the adsorption of CR and CV on the nZVI@nBent–CMC composite was investigated in the temperature range of 298–313 K. In order to understand the nature of the adsorption processes of CR and CV onto the nZVI@nBent–CMC composite, thermodynamic parameters including change in entropy (Δ*S*°), change in enthalpy (Δ*H*°), and change in free energy (Δ*G*°) were computed. The values Δ*H*° and Δ*S*° were determined from the slope and intercept of the van’t Hoff plot, respectively (Figure 7A,B), whileΔ*G*° value is obtained from eq 6
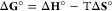
6

The computed thermodynamic parameters are listed in Table 4. For both CR and CV, the Δ*G*° values are negatives at all studied temperatures, confirming the spontaneity of their adsorption processes. For CR, the magnitude of the negative Δ*G*° values decrease with increasing temperature, reflecting higher favorability of the adsorption of CR at lower temperatures. The negative value of Δ*H*° reveals the exothermic process of the adsorption of CR dye onto the nZVI@nBent–CMC composite. In addition, the negative Δ*S*° value manifests the decrease in the randomness at the solid–solution interface during the CR dye adsorption process. For CV, the magnitude of the negative Δ*G*° values increase with increasing temperature, revealing higher favorability of the adsorption of CV at higher temperatures. The positive value of Δ*H*° for CV suggests the endothermic process of the adsorption of CV dye onto the nZVI@nBent–CMC composite, while the positive Δ*S*° value confirms the randomness at the solid–solution interface during the CV dye adsorption process.

**Table 4:** Thermodynamic Parameters for the Adsorption of CR and CV Onto the nZVI@nBent–CMC Composite

dye	temperature (K)	Δ*G*°(kJ/mol)	Δ*H*°(kJ/mol)	Δ*S*° (J/mol K)
CR	298	–11.73	–53.28	–139.43
	303	–11.03		
	308	–10.36		
	313	–9.64		
CV	298	–6.07	38.52	149.65
	303	–6.82		
	308	–7.57		
	313	–8.32		

### 2.6 Adsorption Mechanism

**Figure 8:**
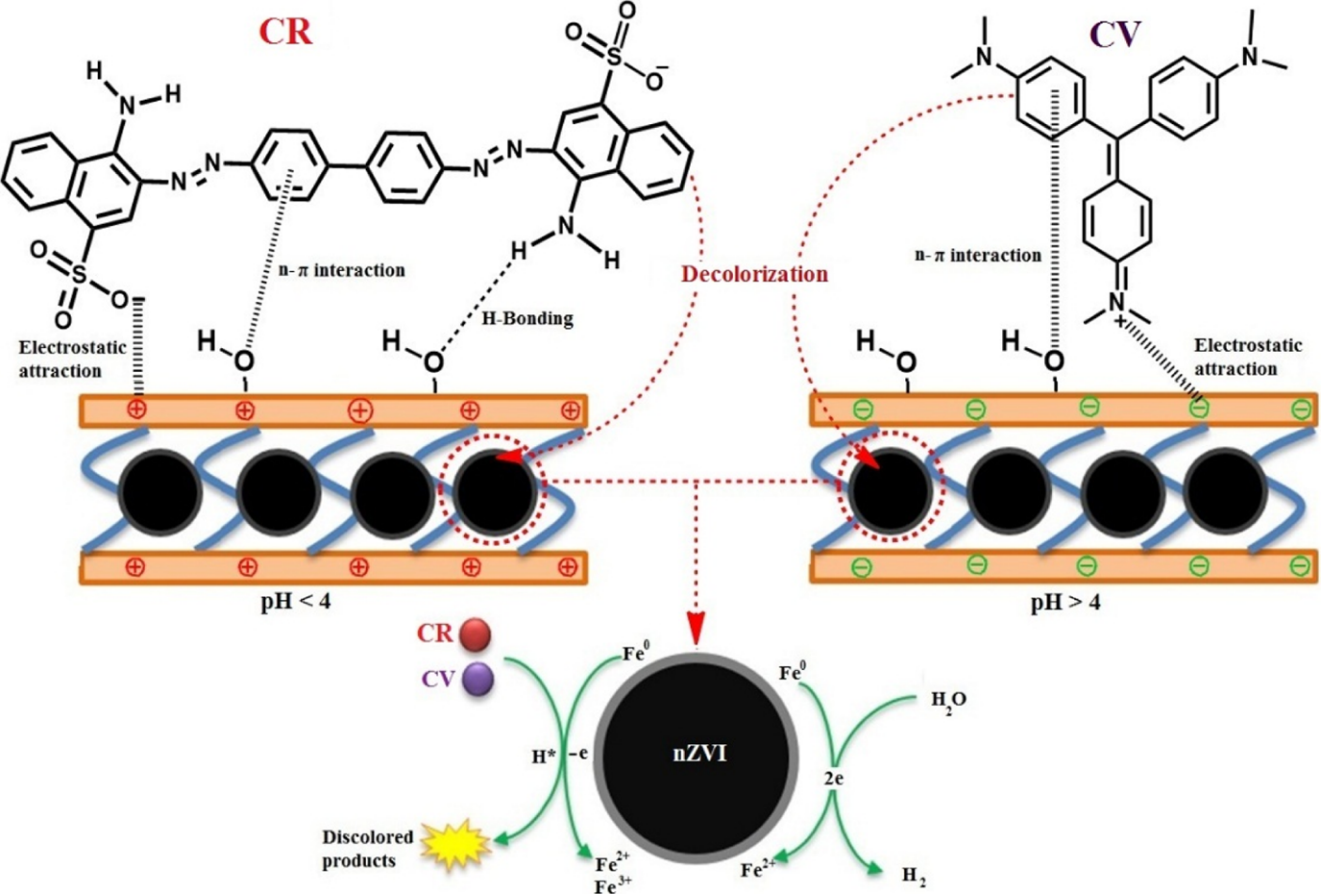
Removal mechanism of CR and CV onto the nZVI@nBent–CMC composite.

The nBent-intercalated CMC play two substantial functions as it prevents the oxidation of Fe^0^ nanoparticles on exposure to air and it provides large surface area by stretching the Fe^0^ chain-like structure. The proposed model for the removal mechanism of both CR and CV onto the nZVI@nBent–CMC composite is depicted in Figure 8. The different removal efficiencies for CR and CV onto the nZVI@nBent–CMC composite could be assigned to the different dye/adsorbent interaction that could be attributed to the molecular structure of each dye. Both CR and CV can interact with the nZVI@nBent–CMC composite surface via electrostatic interaction between the negatively charged CR molecules and the amino groups of the protonated CMC (pH = 2) as well as interaction with metal sites on the nBent surface (i.e., calcium as confirmed by TEM–EDX). However, positively charged CV molecules can attach the negatively charged carboxylate groups of CMC (pH = 6). This indicates the dual functions of CMC at different pH values that enable it to remove both anionic and cationic dyes. Besides, both dyes could form *n*–π interactions between the surface hydroxyl groups of the nZVI@nBent–CMC composite and the benzene rings in each dye. The observed unexpectedly higher uptake capacity of CR onto the nZVI@nBent–CMC composite than that of CV could be explained on the basis of the possibility of H-bonding in the case of CR. However, in the case of CV this H-bonding is not expected because of the presence of tertiary amines and the lack of amine hydrogen atoms.^53^ Another decolorization mechanism for CR and CV on the nZVI@nBent–CMC composite is the reduction of dye molecules via the oxidation of Fe^0^ to Fe^2+^ and/or Fe^3+^ and electron transfer to H^+^ on the adsorbent surface forming atomic hydrogen (H*) and thus decolorization of dyes.^54^

### 2.7 Comparison with Other Reported Adsorbents

The uptake capacity of the as-fabricated nZVI@nBent–CMC composite is compared with different adsorbents reported for the removal of CR and CV, as shown in Table 5. The results clarify that the nZVI@nBent–CMC composite exhibits a greater uptake capacity compared to the reported adsorbents for both dyes.^55−67^ Based on these results, the fabricated nZVI@nBent–CMC composite is a good magnetically bio-based candidate for the removal of both anionic and cationic contaminants from water.

**Table 5:** Comparison of the Maximum Uptake Capacities of CR and CV Onto the nZVI@nBent–CMC Composite With Different Reported Adsorbents

dye	adsorbent	*q*_m_ (mg/g)	reference
CR	nZVI@nBent–CMC composite	884.95	this study
	[poly (Gg-AAm)/ZVI]	250.00	(55)
	BE/CH@Co composite	303.00	(56)
	4A-Cu-300	512.99	(68)
	PVA/MF composite films	221.40	(57)
	AH600-5N	85.00	(58)
	SiMg	78.70	(59)
	ZCAC	83.30	(60)
	carbon composite	298.50	(61)
**CV**	nZVI@nBent–CMC composite	505.05	this study
	chitosan/nanodiopside	104.66	(62)
	MCNCs	333.30	(63)
	SDS-coated MNPs	166.70	(64)
	5G-Fe^0^ NPs/βCD	454.50	(65)
	*Rhodococcus erythropolis* AW3	289.80	(66)
	ZCAC	142.80	(60)
	Keratin nanoparticles	555.50	(67)

### 2.8 Recyclability of the nZVI@nBent–CMC Composite

**Figure 9:**
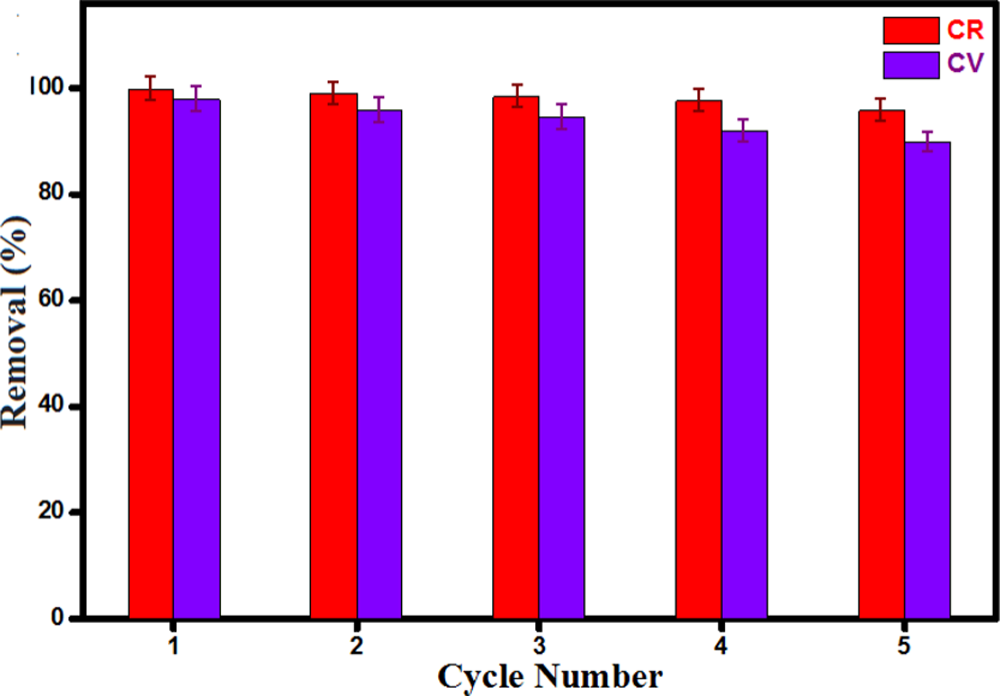
Recyclability of the nZVI@ nBent–CMC composite for CR and CV adsorption [*C*_o_ = 50 mg/L, volume = 100 mL, *m* = 20 mg g, *T* = 25 °C pH_CR_ = 2 and pH_CV_ = 60].

From the industrial point of view, the practical applications not only require the adsorbents that have significant adsorption capacity but also good recyclability. Accordingly, the recyclability of the nZVI@nBent–CMC composite in removal both anionic CR and cationic CV was checked for five sequential cycles. Figure 9 reflects the high removal efficiency of the nZVI@nBent–CMC composite toward the removal of both CR and CV even after five successive cycles. This result can be attributed to the high stability of the synthesized nZVI@nBent–CMC composite as well as its magnetic property that facilitates the separation of the magnetic composite after each cycle without loss in its weight.

## 3 Conclusions

The magnetic bentonite intercalated bio-polymer carboxymethyl chitosan composite was fabricated and tested for the removal of both anionic CR and cationic CV dye using a batch technique. The results revealed that the fabricated composite has an excellent removal efficiency for the anionic CR dye and a very good removal efficiency for the cationic CV dye. Moreover, the adsorption isotherm data showed that the adsorption of CR and CV on the nZVI@nBent–CMC composite could be well described by the Langmuir isotherm with maximum uptake capacities of 884.95 and 505.05 mg/g, respectively. In addition, kinetic data demonstrated that the pseudo-second order is more proper to represent the removal process than the pseudo-first order model. Also, the nZVI@nBent–CMC composite exhibited good reusability and could be considered as an efficient adsorbent for both anionic and cationic contaminants in aqueous solutions.

## 4 Experimental Part

### 4.1 Materials

Chitosan, ferric chloride hexahydrate (FeCl_3_·6H_2_O, 99%), and sodium borohydride (NaBH_4_, 98.7%) were purchased from Sigma-Aldrich. Monochloroacetic acid (ClCH_2_COOH, >99%) and sodium hydroxide (NaOH, 99.5%) were supplied from Loba Chemie. Isopropyl alcohol, methanol, and ethanol were brought from Rankem. Bentonite, crystal violet (CV, C_25_H_30_N_3_Cl, *M*_W_ = 407.98 g/mol, λ_max_ = 598 nm) and Congo red (CR, C_32_H_22_N_6_Na_2_O_6_S_2_, *M*_W_ = 696.66 g/mol, λ_max_ = 494 nm) were obtained from MP Biomedicals.

### 4.2 Preparation of CMC

CMC was prepared according the procedure previously reported in the literature with slight modification.^69^ Exactly, 5 g of chitosan was added to 100 mL of isopropyl alcohol and stirred for 1 h. Then, 60 mL of aqueous NaOH solution (25% w/v) was added to the suspension followed by heating at 70 °C for 2 h. After that, 80 mL of aqueous monochloroacetic acid solution (50% w/v) was added to the reaction mixture slowly over a 15 min period and then kept under stirring for further 4 h. Finally, the product was filtrated, precipitated by methanol, and dried at 50 °C for 10 h.

### 4.3 Preparation of the nZVI@nBent–CMC Composite

The magnetic nZVI@nBent–CMC composite was prepared as follows; at first, 0.1 g of CMC and 0.1 g of nBent were dispersed into 50 mL of deionized water under magnetic stirring for 24 h to obtain a homogeneous nBent–intercalated CMC composite. Then after, 0.2 g of iron(III) chloride was added to the nBent–CMC composite and sonicated for 1 h followed by the dropwise addition of aqueous NaBH_4_ solution (0.3 M, 30 mL), and the mixture was stirred for another 10 min. Finally, the nZVI@nBent–CMC composite was collected by an external magnet, washed with water and ethanol, and dried at 60 °C for 8 h.

### 4.4 Characterization of the Fabricated nZVI@nBent–CMC Composite

The synthesized nZVI@nBent–CMC composite was characterized by XRD (Siemens D-5000) with Cu Kα radiation (λ = 0.154 nm) to identify its crystal phase, while infrared spectra were recorded using a Shimadzu–8400S-Japan. Morphology and elemental analysis were determined by TEM and TEM–EDX, (JEOL-2100 plus TEM), the sample was prepared by sonicating 5 mg of the nZVI@nBent–CMC composite in 10 mL of ethanol for 3 h. Then after, a few drops of the resulting suspension were put onto a grid coated with copper. Furthermore, the magnetism of the samples was determined using a vibrating sample magnetometer (Lake-shore, USA). The specific surface area of the samples was determined using the Brunauer–Emmett–Teller method (BET-Beckman coulter, SA3100, USA). The surface charge of the nZVI@nBent–CMC composite was measured using a Zetasizer (Malvern, UK) because the sample was prepared by dispersing 1 mg of the nZVI@nBent–CMC composite in 10 mL of distilled water, then pH was adjusted by 0.01 M HCL and/or NaOH. Then, the suspension was sonicated for 1 h and injected into the cell of the instrument.

### 4.5 Adsorption Experiments

In this study, CR and CV were chosen as representative anionic and cationic dye models, in order to estimate their adsorption capacity onto the fabricated the nZVI@nBent–CMC composite. Experiments were conducted in batch mode as follow; 100 ml of dye was stirred with 0.02 g of the nZVI@nBent–CMC composite at 25 °C for 60 min at constant stirring speed (150 rpm min^–1^). To study the pH effect, pH was adjusted in the range 2–10 using 0.1 M HCl or NaOH. For adsorption isotherm investigation, 0.02 g of the nZVI@nBent–CMC composite was added to 100 ml of each dye with the initial concentration ranging from 50 to 250 mg/L at 25 °C and optimized pH for interval time 60 min. The effect of temperature and the thermodynamic parameters were estimated by varying the temperature from 25–40 °C. After each experiment, the nZVI@nBent–CMC composite was removed using an external magnet and the amount of unadsorbed dye was measured using spectrophotometry (λ_max_ = 494 nm for CR and λ_max_ = 598 nm for CV). Removal efficiencies (% *R*) and uptake capacities (*q*_e_) of CR and CV onto nZVI@nBent–CMC were calculated as follow
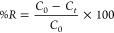
7
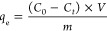
8where, *C*_o_ (mg/L) is the initial dye concentration and *C*_*t*_ (mg/L) is the concentration of residual dye at certain time. *V* (L) is the volume of dye and *m* (g) is the mass of the nZVI@nBent–CMC composite.

All batch experiments were performed in triplicate (*n* = 3), and the obtained data were estimated by the mean value method and correlated by standard deviation (±SD).

### 4.6 Reusability Experiments

To examine the recycling property of the as-fabricated nZVI@nBent–CMC magnetic composite, five adsorption/desorption cycles were executed for both CR and CV. After each adsorption run, the dye-loaded nZVI@nBent–CMC composite was separated by an external magnet and washed with an appropriate eluent (NaCl/methanol solution was utilized to desorb CR, while CV was desorbed by absolute ethanol). Next, the recycled nZVI@nBent–CMC composite was dried at 50 °C for 6 h and then utilized in the following cycle.
